# Defining Wider Indications for Stoppa Repair Other Than Recurrent Hernias

**DOI:** 10.7759/cureus.23671

**Published:** 2022-03-31

**Authors:** Juwairiah Abdur Raheem, Suresh C Annu, Rabiya Begum, Hafsa Iqbal, Abdul Majid A Mohammad

**Affiliations:** 1 Department of General Surgery, Deccan College of Medical Sciences, Hyderabad, IND; 2 Department of Surgical Gastroenterology, Deccan College of Medical Sciences, Hyderabad, IND; 3 Department of General Surgery, Telangana Institute of Medical Sciences, Hyderabad, IND; 4 Department of Urology, Deccan College of Medical Sciences, Hyderabad, IND

**Keywords:** stoppa repair, giant hernia, groin hernia, preperitoneal mesh placement, giant prosthetic reinforcement of the visceral sac, abdominal hernia

## Abstract

Managing complex inguinal hernias has been a constant challenge for surgeons and its treatment is not without challenges with the routine current techniques. Complex inguinal hernias especially recurrent have been managed by the Rives-Stoppa technique which is an established suture-less, tension-free, and absolute method of treatment with minimal recurrence rates. Traditionally, this surgical technique is most indicated in recurrent inguinal hernias, but we aim to assess the usefulness of this procedure for the treatment of complex inguinal hernias in individuals presenting for the first time. We report four varied cases of complex inguinal hernias, repaired by the open Rives-Stoppa technique, and discuss its indications, technique of repair, and current status.

## Introduction

Complex inguinal hernias of the abdominal wall constitute an important public health problem and often pose a surgical dilemma even for the most skilled surgeons [1]. Complex inguinal hernias constitute recurrent inguinal hernias, giant inguinal hernias wherein more than 50% viscera lie in the hernial sac as the scrotal abdomen, bilateral inguinal hernias with giant component or wide deep inguinal ring (more than 7 cm) or large inguinal hernias associated with other types of infra-umbilical abdominal or pelvic internal hernia. Large lower abdominal hernias are defined as defects located 3 cm above the pubic symphysis to the umbilicus and are larger than 10 cm in diameter [2]. The high incidence of the disease makes hernia repair the most frequently performed procedure in general surgery, accounting for 10% to 15% of all operations [3]. In order to treat these complex inguinal hernias, it is mandatory to adopt an alternative approach to reduce its complications [4,5]. Usually, the traditional anterior approach by Bassini or Lichtenstein techniques employs mesh about 12/15 cm, which would be inadequate to cover giant inguinal hernial neck diameters more than 8 cm, so that there is at least 5 cm overlap all round inguinal sac neck. Even placing a 15/15 cm mesh would have technical difficulties to get adequate overlap, especially in the inferior part of the deep ring causing persistent weakness in the myopectineal orifice of Fruchard. Hence adopting an alternate, to reach the myopectineal region by posterior pre-peritoneal approach would help and, in this regard, the Rives-Stoppa technique achieves both an anatomic and a prosthetic repair reinforcing not only the myopectineal region but the whole of the infra-umbilical abdominal wall [5]. The Stoppa procedure also called giant prosthetic reinforcement of the visceral sac (GPRVS) is performed by wrapping the lower part of the parietal peritoneum with a mesh. The mesh contributes to a physiological healing process and creates a special bilateral anatomical reinforcement in whole of infra-umbilical and inguinal/myopectenial region which effectively prevents hernia recurrence, whereas traditional anterior inguinal and ventral hernia mesh repair may fail in achieving these goals. GPRVS is usually performed in patients fit for general anaesthesia, in recurrent hernias (repaired prior by anterior repairs), or large and/or bilateral inguinoscrotal hernias. It requires a wide dissection of subfascial preperitoneal space. The rationale is based on elegant surgical and anatomical prosthetic placement that occludes myopectineal ostium of Fruchard for inguinal and tenseless mesh placement for ventral hernia, especially infra-umbilical region.

An open approach to repair complex inguinal hernia and those associated with multiple hernias especially in the lower abdomen needs special procedure like Stoppa where the mesh is placed in a potentially created preperitoneal space reinforcing the whole of the lower visceral peritoneal sac.

## Case presentation

We report four cases of giant lower abdominal hernias (Table 1) operated by the Rives-Stoppa technique.

**Table 1:** Summary of all cases

Case	Age (years)	Sex	Comorbidities	Stay in hospital (days)	ICU stay (days)	Neck diameter (centimetres)	Hernia size (Cranio-caudal length ×antero-posterior length× medio-lateral length in cm)	Final diagnosis	Outcome	Follow up duration (months)
1	52	M	None	7	2	13	30×20×10	Large inguinal scrotal hernia with enterocele	Uneventful	24
2	85	F	None	7	Not required	11	14×14×10	Right sided obstructive inguino-labial hernia along with procidentia	Uneventful except for seroma	24
3	32	M	Morbidly obese (BMI 35)	5	Not required	8	10×10; 8×10; right and left inguinal; umbilical 9×9; urinary diverticulum 12×10×10	Bilateral inguinal and umbilical hernia having urinary bladder as sliding component	Uneventful	18
4	55	M	None	8	2	13	30×20×10	Bilateral inguinal hernia, right side scrotal abdomen with left side moderate hernia	Uneventful	12

Case 1

**Figure 1:**
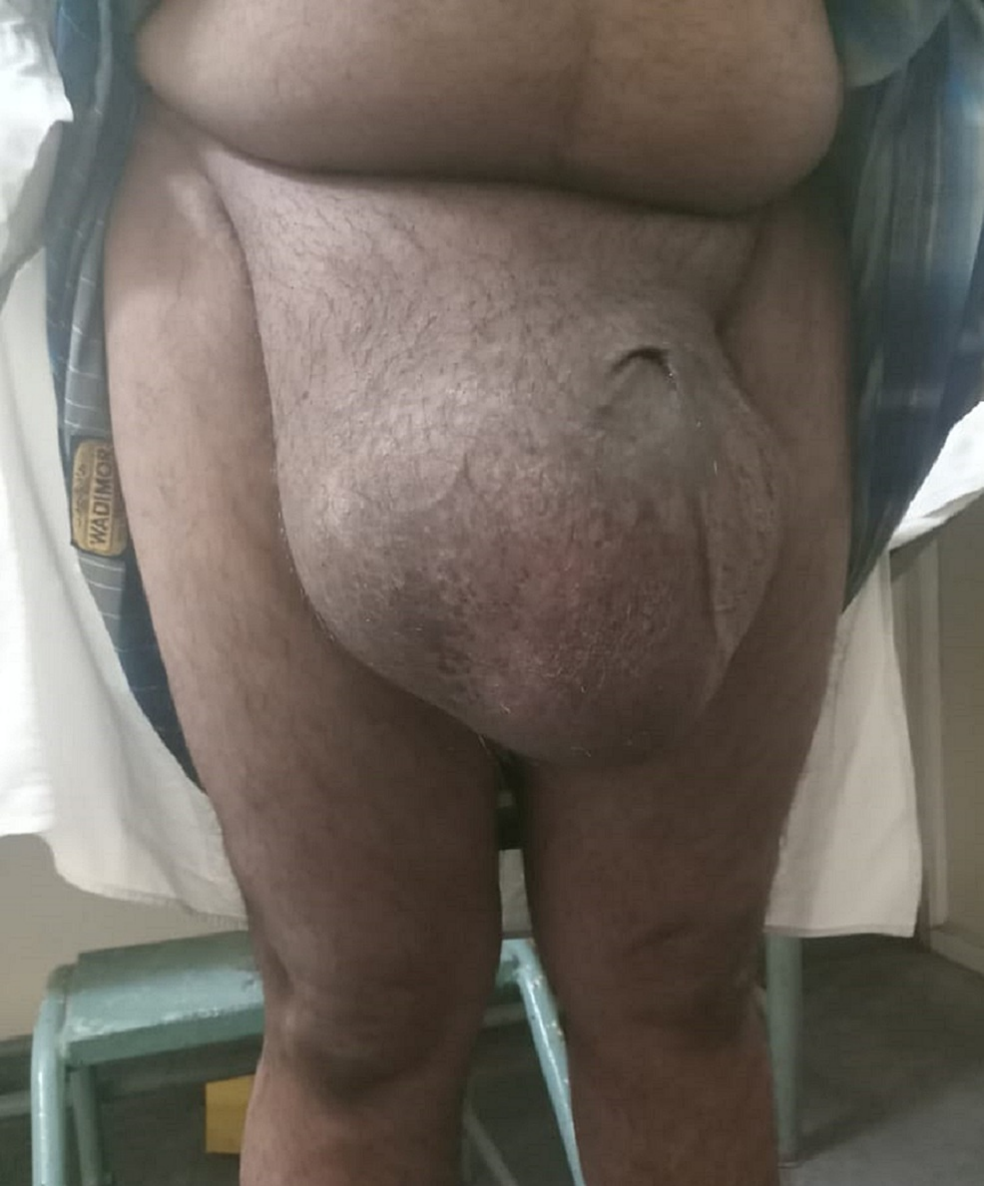
Pre-operative scrotal abdomen

A 52-year-old fit gentleman presented to the surgical department with a giant inguinoscrotal swelling for more than a decade. On examination, it was found to be a large, non-reducible right inguinal hernia with penis buried in the skin and invisible (Figure 1).

**Figure 2:**
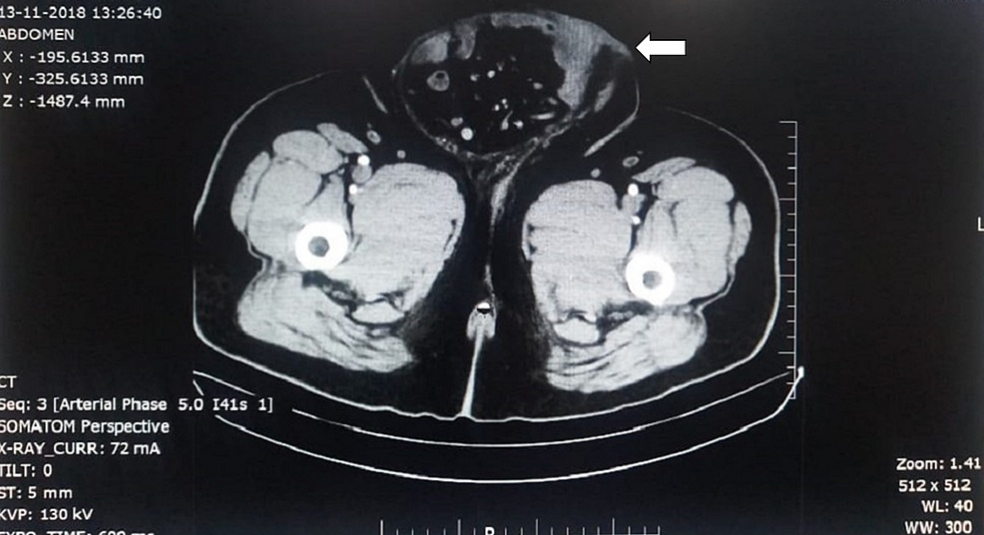
CECT abdomen showing large inguinal scrotal hernia with enterocele at mid-thigh level CECT- Contract-enhanced computed tomography

His routine haematological and biochemical investigations were unremarkable. Contrast-enhanced computed tomography (CECT) abdomen (Figure 2) revealed a large inguinal scrotal hernia with omento-enterocele (40%-50% of intestines in the scrotum).

**Figure 3:**
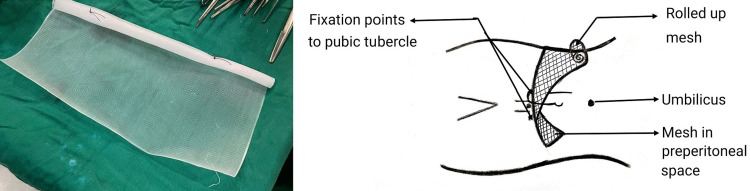
Rolled up giant mesh placement and fixation

**Figure 4:**
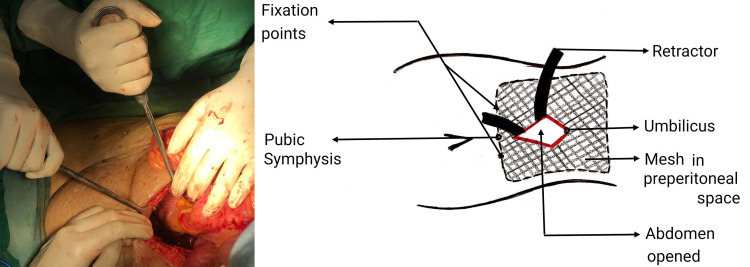
Mesh spread in the retropubic space of Retzius

As a large portion of the abdominal contents were in the scrotum, it was decided to proceed with a lower midline incision. Pre-operative prophylaxis for abdominal compartment syndromes like incentive spirometry and deep breathing exercises with contents as reducible as possible, was started six to eight weeks prior to the date of surgery. Intra-operative findings revealed large hernial sac with wide neck, 13 cm containing ascending colon, cecum, ileocecal junction, appendix and small bowel loops with dense intra-sac adhesions. After contents reduced and sac excised, a half-rolled mesh was placed in extravesical space after dissecting the preperitoneal space. The mesh (size 30cm×30cm) was spread in retropubic space of Retzius adjacent to the pubic symphysis on both sides with mesh being fixed to both tubercles with No.1 polypropylene suture (Figures 3, 4). The post-operative period was uneventful. The patient was admitted to the intensive care unit for two days with respiratory support for anticipated abdominal compartment syndrome.

Case 2

**Figure 5:**
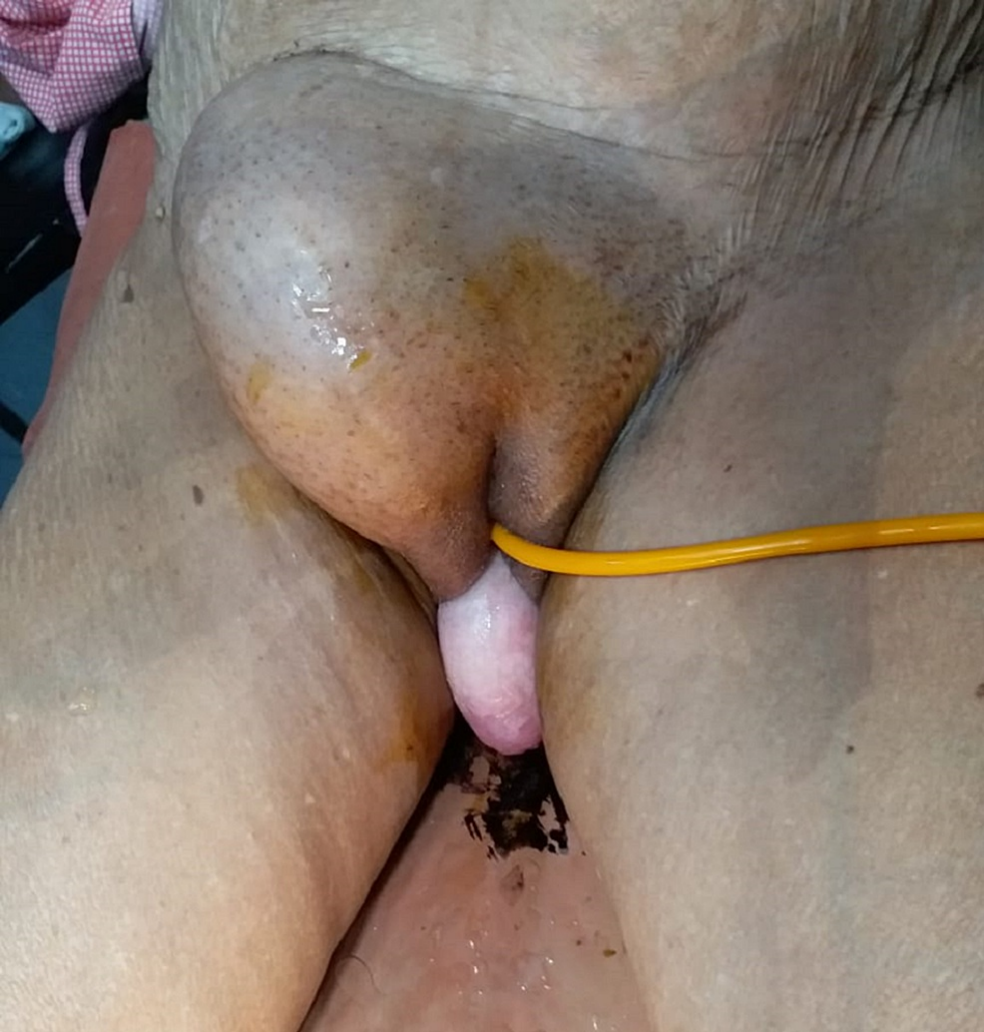
Large obstructed inguino-labial hernia along with procidentia

An 85-year-old lady with no comorbidities presented to the surgical department with right-sided obstructive inguino-labial hernia along with procidentia (Figure 5).

After pre-operative resuscitation and stabilization, the abdomen was explored with a lower midline incision. Intra-operatively, a defect of 11 cm with sac of size 14×14×10 cm containing terminal ileum, ileocecal junction, ascending colon, transverse colon, transverse mesocolon and greater omentum as contents. Adhesiolysis allowed the contents to get reduced and aided in sac excision. Mesh was placed in preperitoneal space and fixed to both pubic symphyses. During the post-operative period, she developed seroma, which was managed conservatively with only pressure dressings and was discharged on post-operative day 7. Here, a midline approach helped to assess bowel viability, adhesiolysis, reduction of contents, and placement of giant mesh to reinforce both sides of myopectineal orifices.

Case 3

**Figure 6:**
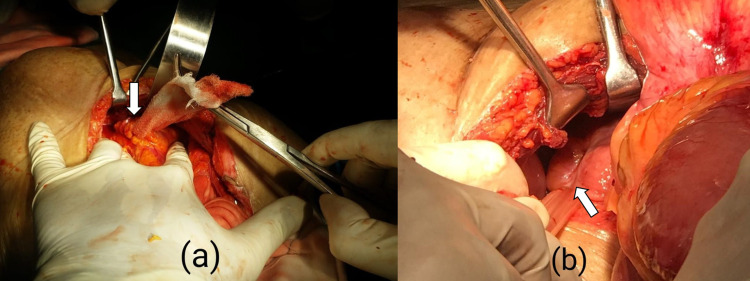
Arrows showing (a) left cord with contents isolated and (b) urinary bladder as sliding content mimicking as bladder diverticulum

A 32-year-old gentleman with morbid obesity (BMI 35), presented to the urology department with increased frequency of micturition. On clinical examination, bilateral inguinal and umbilical hernias were observed. CECT abdomen revealed bilateral inguinal and umbilical hernia with urinary bladder diverticulum. Uro-dynamic studies ruled out bladder outlet obstruction. Other pre-operative investigations were unremarkable. To address these three different pathologies, a lower midline incision was taken which helped in having a single incision along with the simultaneous treatment of differently located hernias and further revealed the exact aetiology of left inguinal hernia having urinary bladder as sliding component, which was mimicking bladder diverticulum on pre-operative CT imaging and aided in intraoperative giant mesh placement in preperitoneal space (Figures 6a, 6b). The immediate post-operative period was uneventful, so also follow-up of 18 months.

Case 4

**Figure 7:**
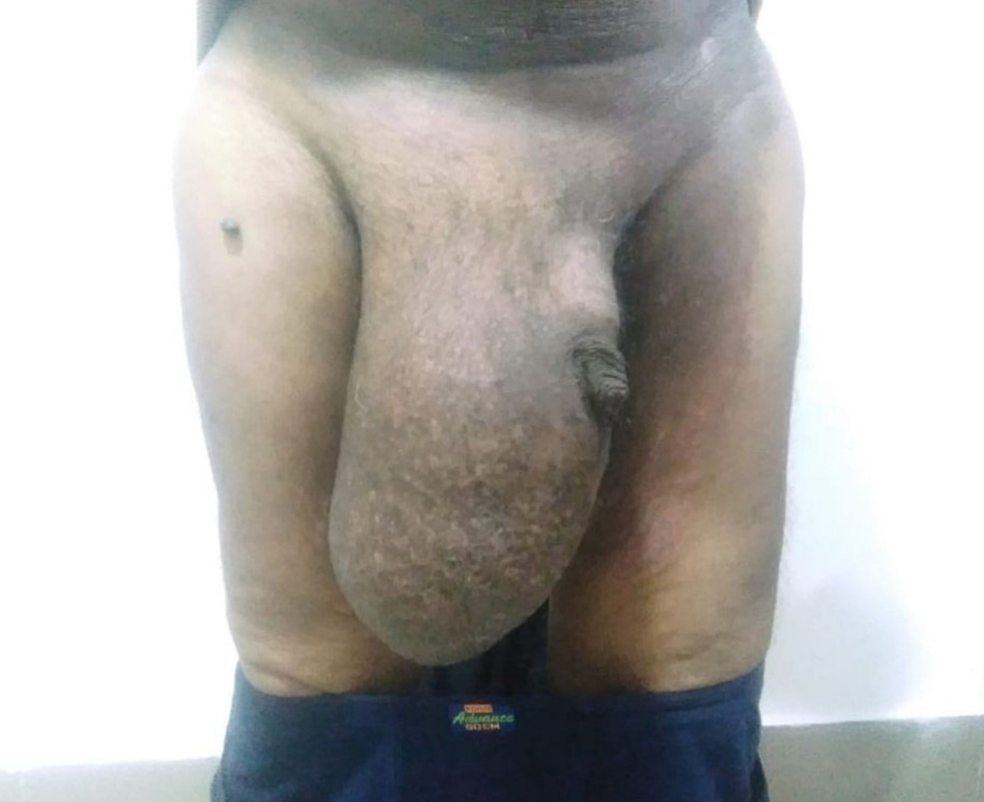
Right inguinal hernia presenting as the scrotal abdomen

A 55-year-old gentleman with no comorbidities, presented to the surgical department with a bilateral gradually progressive hernia for the past 18 years disabling him to walk due to right-side hernia being giant/scrotal abdomen reaching almost to the knee level and left side moderate size hernia (Figure 7).

**Figure 8:**
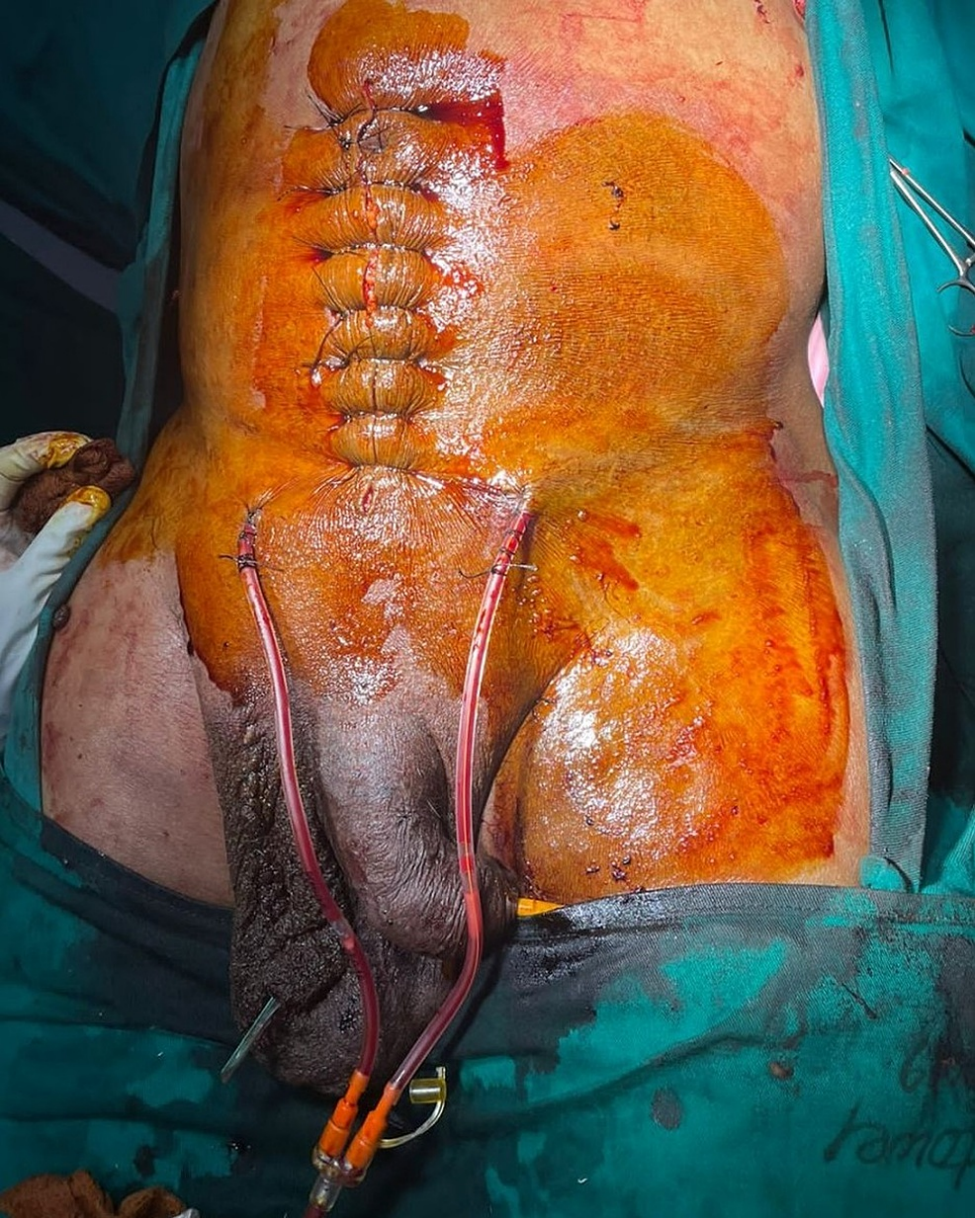
Lower midline incision with abdominal suction drains in retro-pubic space and scrotal incision with scrotal corrugated drain

After pre-operative evaluation, to check fitness for anaesthesia and predisposing causes for hernia (chronic cough or obstructive uropathy), patient was prepared for surgery by prior pulmonary exercises and incentive spirometry for six to eight weeks to improve his Forced Expiratory Volume in the first second. Preperitoneal mesh repair was done with lower midline incision, which revealed 13 cm wide neck sac containing small bowel loops, greater omentum, caecum, ileo-colic junction, ascending colon and hepatic flexure as contents. A scrotal incision required, as the contents were firmly adherent to the sac and were not reducing by abdominal approach (Figure 8). After adhesiolysis, the contents were reduced. Post-operatively, the patient required positive ventilatory support for two days due to transient abdomen compartment syndrome.

## Discussion

In the era of minimal access surgery (MAS) gaining popularity to replace traditional open access approaches, there is still a sub-group of complex inguinal hernias presenting for the first time, which might best be managed by open preperitoneal access because of various technical challenges in MAS or anterior open approaches. Dense intra-sac adhesions would impede reduction during MAS whereas, the relatively smaller mesh would be insufficient to place in an inguinal canal to re-enforce the lower abdominal wall and myopectineal region by open anterior approach, especially for giant or complex inguinal hernias. Stoppa offers good results with a minimum chance of recurrence [6].

Patients planned for Stoppa repair, require pre-operative abdominal and chest exercises using three-ball incentive spirometry, three to four weeks prior to surgery to increase abdominal and pulmonary reserve capacity. This helps in faster post-operative recovery and reduces the risk of abdominal compartment syndrome and the need for ventilatory support. A single lower midline incision helps to simultaneously address multiple hernial sites, dense intra-sac adhesions, and in an emergency to tackle obstruction or strangulation. Post-operatively epidural analgesia would be particularly helpful not only for adequate analgesia but also to keep the abdominal muscles relaxed and acclimatize the abdominal cavity for extra visceral accommodation after hernia reduction. In a way, epidural analgesia also reduces the risk of renal hypoperfusion, avoiding nephrotoxic analgesia, which is bad consequences in case there sets in increased intra-abdominal pressure risking abdominal compartment syndrome. A post-operative bed with ventilatory support for the first 24-48 hours should be reserved for emergent management of abdominal compartment syndrome, in case it develops. Seroma is not an uncommon complication with a recorded risk of 21.4% [7], comparable to noted herein of 25%, which is mostly managed conservatively by pressure dressings.

**Figure 9:**
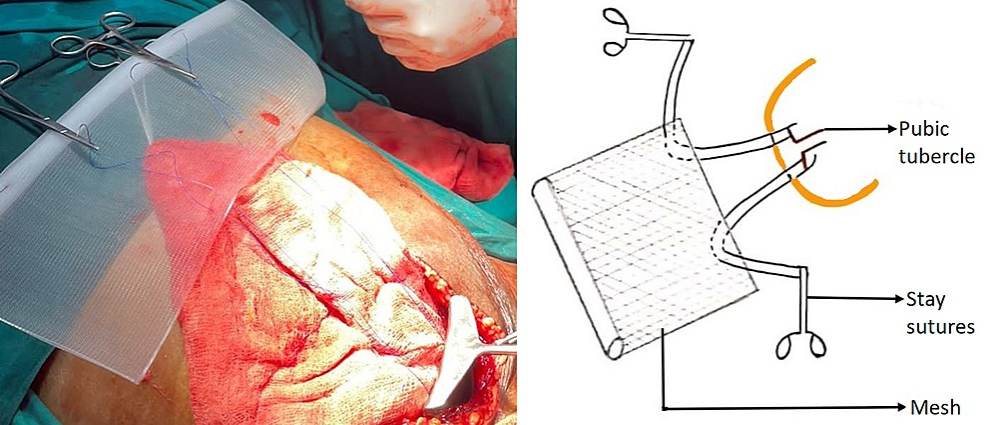
Preparation of mesh

The Stoppa technique is the most promising open technique, with low recurrence rates, excellent long-time results, and minimal serious morbidity [8,9]. It seals the inguinal, femoral, and obturator canals (myopectineal orifices) as well as all other potential sites of weakness in the lower abdomen which makes late recurrence unlikely [10]. It has met success in the repair of bilateral, recurrent, large scrotal, and obstructed hernias (with viable bowel and less oedema of bowel loops) in which conventional repair is difficult and carries high morbidity and failure rates. In this procedure, the lower part of the parietal peritoneum is wrapped with a giant prosthetic mesh [11]. Fixation of mesh is debatable for small or medium hernia repairs, but for large or giant complex hernias, placement of mesh with fixation would help in intra-procedure manipulation for its proper alignment (Figure 9).

Advantages of Stoppa technique

**Figure 10:**
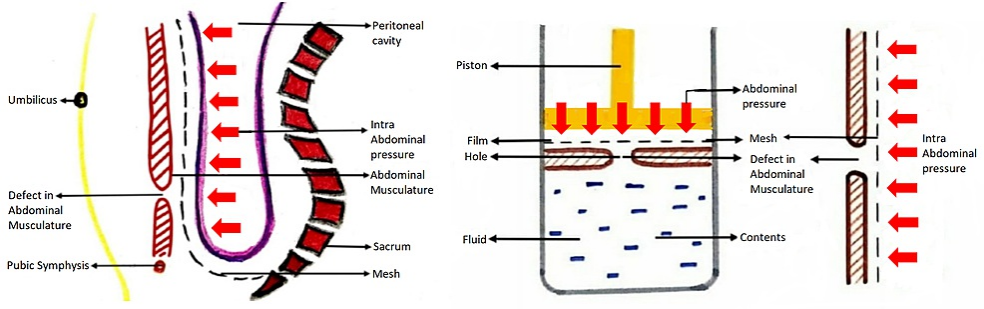
Sagittal section of abdomen and pelvic cavity representing sublay mesh repair along with schematic representation of Pascals principle

The procedure uses a midline incision and avoids reopening through scarred anatomy in cases of recurrent inguinal hernias and also provides convenient access for repair in non-recurrent hernias like giant inguinal scrotal hernias, large bilateral inguinal hernias, wide deep inguinal ring more than 8 cm in diameter, medium to large obstructed/strangulated inguinal hernias and in unilateral scrotal abdomen. It exploits Pascal's principle to hold the mesh without tension (Figure 10).

**Figure 11:**
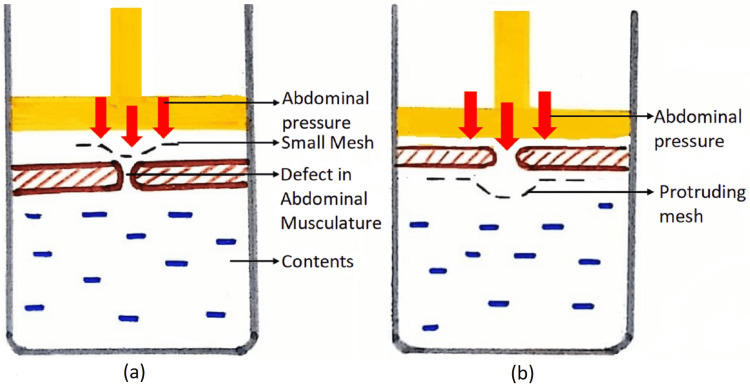
(a) Sublay mesh repair with small mesh showing increased risk of herniation (b) Onlay mesh repair, depicting increased risk of recurrence

A large preperitoneal mesh is used, as a small mesh when placed in the preperitoneal space will protrude through the defect when the abdominal pressure increases (Figure 11a) whereas a large mesh will be held in position by the same abdominal pressure exploiting the pascals principle. The mesh in onlay repair is constantly exposed to intra-abdominal pressure through the same hernial defect and increases the chance of recurrence of hernia (Figure 11b).

Sublay mesh repair has been proven to be superior to onlay mesh repair [12-14]. A meta-analysis showed inconclusive recurrence rates in comparison of the two types of hernial mesh repair [15] whereas, Venclauskas et al. concluded a recurrence rate of 10.5% by onlay mesh repair and 2% using sublay mesh repair [16], which satisfactorily proves the adequate application of physical forces as highlighted in our study. Stoppa allows for the inspection of all potential abdominal hernial sites, not only the inguinal region but also the complete myopectineal orifice of Fruchaud of both sides and any other ventral hernias (umbilical, infraumbilical incisional hernia). The risk of nerve injury, neuralgia, orchitis, testicular atrophy and chronic neuropathic pain is reduced via this technique. From the patient's perspective, self-confidence and a better aesthetic result are added advantages. The patient can void urine properly, as he was unable to do so, prior to surgery due to large scrotal hernia embedding the penial urethra.

Limitations of Stoppa technique

Mostly performed under general anaesthesia and requires midline incision remote from the site of primary hernia. It requires meticulous dissection, for potential preperitoneal space creation, which can increase the attending risk of seroma by 25% as noted and has a remote chance of enterotomy thereby precluding the very purpose of mesh repair. Post-operative analgesia required is more in comparison to other hernial repairs.

## Conclusions

Even in this current era of minimal access, surgery is established as the standard of care in managing all types of complex abdominal wall hernias. We have tried to identify a subset of complex infra-umbilical hernias, other than routinely indicated recurrent hernias to select Open Stoppa repair because of inherent technical challenges with all present procedures. We hope to see this traditional technique continues to be in surgeons' armamentarium even in patients presenting for the first time with complex/giant infra-umbilical inguinal hernia and helps reinforce tension-free mesh repair not only in the region of myopectineal orifice of Fruchard but also the whole of infra-umbilical abdominal wall exploiting Pascal’s principle.
